# Sustainability assessment as problem structuring: three typical ways

**DOI:** 10.1007/s11625-016-0417-x

**Published:** 2017-01-10

**Authors:** M. Dijk, J. de Kraker, A. van Zeijl-Rozema, H. van Lente, C. Beumer, S. Beemsterboer, P. Valkering

**Affiliations:** International Center for Integrated Assessment and Sustainable Development (ICIS), Maastricht University, P.O. Box 616, 6200 MD Maastricht, The Netherlands

**Keywords:** Sustainability assessment, Assessment approach, Problem structuring, Reflexivity

## Abstract

Sustainability assessment (SA) is an increasingly popular term referring to a broad range of approaches to align decision-making with the principles of sustainability. Nevertheless, in public and private sectors sustainability results are still disappointing, and this paper reflects on this problem and proposes a way forward. We argue that, because sustainability issues are generally wicked problems (i.e. a ‘complex of interconnected factors in a pluralistic context’), effective assessments need to be reflexive about the definition of the issue and about the criteria for sustainable solutions. Based on a distinction of policy problems, we characterize SA as a form of problem structuring, and we distinguish three typical ways of problem structuring, corresponding to three different ways of integrating reflexivity in the assessment. We illustrate these routes in three examples. We discuss the way reflexivity is integrated in each example by discussing the mix of methods, SA process and epistemological balance. Rather than merely calling for more stakeholder participation, our aim is to call for more reflexivity integrated into the SA approach, and we conclude by proposing a process map for reflexive sustainability assessment to support this.

## Introduction

Sustainability assessment emerged as a marriage between environmental assessment and sustainable development (Gibson et al. 2005). Sustainability assessment (SA) is nowadays a widely used term that covers a broad range of approaches aiming to operationalize sustainability concepts for decision-making, mostly within but also outside governments. These approaches may be formal or informal, legally prescribed or voluntary, science-driven or policy-driven, etc., and may carry different labels, such as sustainability appraisal, sustainability impact assessment or integrated assessment (Pope 2006). A common feature is that they try to integrate various perspectives, interests, and types of knowledge. However, despite scholarly progress, Gibson (2016) concludes that in public and private sectors disappointing little has been accomplished on the sustainability front in the last decade. He suggests that the main reason for limited progress is that the comprehensive, integrative and open approach of SA fits poorly with the entrenched structures, cultures and motivations of conventional authorities. In this paper we reflect on the poor results in the practice of decision-making further by critically examining the SA approach, focusing especially the extent to which the process of defining the issue and the criteria for sustainable solutions is explicit and integrated in the assessment.

Sustainability issues are generally ‘wicked’ problems (Rittel and Weber 1973). They are wicked in the sense that they are characterized by a complex of interconnected factors as well as a pluralistic context, implying that they (1) can be defined and explained in numerous ways, (2) are unique, (3) are connected to other problems, and (4) do not have a single, objectively best, definitive solution or a well-described procedure to find a limited set of potential solutions (ibid.). We will argue that this ‘wicked’ nature implies that effective assessments need to be reflexive about the definition of the issue and about the criteria for sustainable solutions.

Drawing on a basic distinction in policy studies, between structured and unstructured problems we characterize SA as a way to structure policy problems, and we distinguish three typical ways to structure sustainability problems.^1^ Rather than merely calling for more stakeholder participation, our aim is to call for more reflexivity integrated into the SA approach that will lead to more effective recommendations for decision-making in practice.

This paper is structured as follows: In the next section, we review the key traits of SA, including the critiques it has received, subsequently we argue that every SA can be seen as a form of problem structuring and we identify three typical ways of reducing the complexity of problems encountered in SAs. An illustrative example of each way is provided in the subsequent section. Finally, we discuss the challenge to integrate more reflexivity into SA by discussing ways to align the mix of methods, process design, and epistemological balance and we conclude by proposing a process map for reflexive sustainability assessment to support this.

This paper builds on earlier pleas for more reflexivity. These, however, were emphatic at a more general level (e.g. Voß et al. 2006 on reflexive governance for sustainable development and Rayner and Malone 1998 on combining positivist and interpretative approaches in the global climate change debate), and less elaborated at project or case level. This paper offers an important, first step towards an operationalization of this plea at the level of a particular assessment project.

## Key traits and critiques on SA

The seminal contribution of Gibson et al. (2005) presents criteria and processes to understand the context and strategies for sustainability assessment. It especially emphasizes the process of formulating criteria through a number of steps, including formulation of case and context considerations, criteria specification, categorization, and elaboration (including development of trade-off rules, identification and evaluation of alternatives). Gibson (2016) focuses on applying this understanding by discussing a large variety of cases. The book shows the challenges of applying generic criteria and trade-off rules in the peculiarities of each context.

The ambition to operationalize sustainability for decision-making and the wicked nature of sustainability issues (i.e. generally a ‘complex of interconnected factors in a pluralistic context’) have implications for the knowledge questions that should be addressed in an SA. It raises two distinct types of knowledge questions. Questions regarding the physical and monetary impacts, such as: what sorts of substances are emitted, what materials are used, and what waste is produced (e.g. Sander and Murthy 2010)? What are consequences for economic indicators such as cost (e.g. Ekins and Vanner 2007)? These are questions about causes and effects. Secondly, there are normative questions related to stakeholder interpretations, such as: what are different stakeholder perceptions of the issue (e.g. Setiawan and Cuppen 2013)? What is their contextual definition of ‘sustainable’? These are questions about priorities, values and desirability. This means that for an assessment to be effective, it needs to address both types of questions, and hence be reflexive about the definition of the issue and about the criteria for sustainable solutions. ‘Reflexive’ then refers not only to the special attention for how multiple stakeholders define the issue and criteria for solutions, but also for how the step from these different frames to the particular definition of issue and criteria applied during the assessment was made.

SAs in practice have been challenged to deal with both types of questions, and, accordingly, have received broadly two types of critique. Some scholars argue that integration of a wider range of stakeholders’ perspectives into assessment remains rather limited (Turnpenny et al. 2008). Stakeholder involvement, they argue, is often restricted to providing input on the choice between a limited set of options, rather than radically redirecting policy.^2^ These issues may relate to lack of problem awareness among stakeholders, unbalanced problem ownership or discontinuous participation (Lang et al. 2012). Other scholars argue that guidance on what methods can be used in sustainability assessment is lacking, since guidelines (e.g. for an environmental impact assessment) are typically biased towards certain procedural steps (De Ridder et al. 2007). The research on how to organize and deploy tools and methods in assessments has a lot of room for improvement (Wiek et al. 2012). Also, in collaborative assessments of scientists and practitioners, it can be difficult to agree on the methodological standards (Lang et al. 2012). In the following we unpack the two types of critique.

### Mix of methods and process design

A first cross-cutting critique concerns the question what methods to use and how to organise the SA process. While method and process are clearly linked, this refers to two separate questions: how can the results of various methods applied in an SA be combined and how can the process be organized?

#### Process design

An SA is designed to form a logic sequence within an analytic and decision-making process, and within which a range of different methods can be applied (Finnveden et al. 2003). There is no single and commonly accepted procedure for sustainability assessment. A procedure may be formally prescribed by law, such as in environmental impact assessment (EIA) in many countries (but with great variety between countries) and as strategic environmental assessment under the EU SEA Directive (2001/42/EC). In the case of non-legally prescribed, science-driven SA, the procedure is of course up to the researchers. Many different descriptions of the steps or phases of integrated assessment can be found in the literature.^3^ Finnveden et al. (2003), for instance, apply (1) definition of objectives, (2) formulation of alternatives, (3) scenario analysis, (4) environmental analysis, (5) valuation, (6) conclusions and follow-up. Norse and Tschirley (2000) apply (1) problem identification, (2) strategy formulation, (3) selection of policy options, (4) policy implementation, (5) setting of regulatory standards, (6) monitoring and evaluation. Sheate et al. (2003) suggest screening, scoping, baseline survey, evaluate impacts, report, monitor and review, while Lang et al. (2012) use (A) collaborative problem framing and building a collaborative research team; (B) co-creation of solution-oriented and transferable knowledge through collaborative research; (C) (re-) integrating and applying the co-created knowledge. De Ridder et al. (2007), however, show there are strong complementarities and overlaps between the different assessment procedures since they share the same origin in policy analysis (Dunn 2003; Hogwood and Gunn 1984) and systems analysis (Miser and Quade 1985; Quade 1983). Therefore, De Ridder et al. map different assessment procedures (for example IA, EIA, SEA) onto a more basic, or generic, procedure consisting of four steps or phases: (I) problem analysis, (II) finding options, (III) analysis of options and (IV) follow-up.^4^

#### Mix of methods

A broad range of methods has been applied in SA, with often combinations of methods being used within one study. However, in assessments in the public and private sector, the choice is often poorly explained and, when combining methods, often one method is clearly dominant and basically shapes the SA outcomes (of which we provide examples in “Examples of the three ways of problem structuring”). A common problem identified in the literature is the lack of guidance on what methods can be used (Noble et al. 2012), since guidelines are typically biased towards certain procedural steps (De Ridder et al. 2007). Research on how to organize and deploy tools and methods in assessments seems to have a lot of room for improvement (Wrisberg et al. 2002; Nilsson et al. 2005; Lee 2006; Wiek et al. 2012).

Van Asselt (2000) distinguishes two groups of methods: participatory methods and analytical methods, yielding, respectively, subjective knowledge elements (referred to as ‘the value-laden information provided by societal actors’) and objectified knowledge elements (referred to as ‘scientific facts’). Rotmans (2001) argues for a combination of these two types of methods. In his view, scientists can provide the latter on their own, but should engage societal stakeholders to deliver the first. Subsequently, the actor perspectives and the findings from analytical methods should come together in an active dialogue (Rotmans 2001, p.22). Hence, he views integration as a dialogue or participatory process, without being too explicit on how this can be done.

De Ridder et al. (2007) build upon Van Asselt and Rotmans’ work and distinguish, apart from participatory methods, additional sub-groups of analytical methods: scenario tools, multi-criteria analysis tools (MCA), cost-benefit analysis (CBA) and cost-effectiveness analysis (CEA), accounting tools, physical analysis tools and indicator sets, and modelling tools. Again, the question how to combine these is only generally addressed (Bond et al. 2011).

De Ridder et al. (2007) also address the linkages between the mix of methods and procedural steps, although they are not explicit on how results from different tools should be combined. They discuss which methods are more applicable in which phase and suggest that participatory tools are more appropriate in the first phase of the SA, the problem analysis, to elucidate views of the various stakeholders, explicate knowledge and values at stake, and ideas about ways to alleviate the problem. In the two subsequent phases (i.e. identification and analysis of various options), when the focus has crystallized, analytical tools are more appropriate. In the final phase, follow-up, participatory tools are emphasized again, this time as an appropriate means to evaluate the outcomes and assessment process.

### Epistemological balance

The critique that SAs have difficulties to integrate stakeholder perspectives is also related to Rayner and Malone’s plea for a better integration of positivist and interpretative approaches. Science is not a monolithic affair but contains various traditions, approaches and methods, which, in their turn, are rooted in diverging ontological and epistemological assumptions. They tend to progress alongside each other, but in cases like sustainability problems this divergence is problematic. This is not a new insight. For instance, with reference to Snow’s (1959) classic essay on ‘two cultures’, Rayner and Malone (1998) expound the two styles of science that characterize the intellectual landscape since the 18th century: the natural scientific approach with a descriptive or positivistic approach versus the humanities with an interpretative approach.^5^ They see the isolated existence of these two, primarily epistemologically different approaches as problematic—not their co-existence as such. Hence, they argue that especially climate change issues can benefit from a combination of the two. Although other epistemological categorizations have been proposed (see for instance Morgan and Smircich 1980 who suggests a range of epistemological stances within a positivist to anti-positivist dichotomy^6^), in the following we summarize Rayner and Malone’s exposition because it illustrates the epistemological split most clearly.

The positivist approaches operate from the assumption that the world consists of entities, which are just “out there” and of which knowledge can be obtained by measuring them. The implication of this assumption is that in the case of different ideas of what happens in the world, the route to conciliation is to provide better data, better models and better ways to integrate them. Interpretative approaches, on the other hand, emphasize that ideas on what happens in the world are always mediated by interpretative schemes, such as language, concepts, and routines. Data will never speak for themselves, but can only be meaningful and plausible when accompanied and supported by socialization, training, and practices of interpretation. As a consequence, when ideas on what happens in the world differ, the route to conciliation is to become reflexive about interpretative schemes and to discuss and articulate what makes the ideas meaningful and plausible.

The interpretative method focuses on understanding the meaning that human agents create during the conduct of social life, upon which they build their understanding of the world, and through which they seek to act upon that world. Thus, the interpretative method focuses on the nature of experience, the structure of perceptions, the recognition of interests and the development of frameworks of collective action. The systematic study of the way issues or problems are framed yields understanding that can help decision makers make critical choices, knowing what assumptions and decision elements underlie those choices (Bacchi 2009).

The distinction between the two approaches raises fundamental issues of what kinds and sources of knowledge the analyst values. Does (s)he want to understand the issue as seen through the eyes of stakeholders (interpretative) or does he value ‘objective’ observation (positivist)? We refer to the ‘epistemological balance’ of an assessment as the balance between these two different kinds and sources of knowledge.

The two kinds of approaches have long lived as two fields separated from each other. This is problematic, because, instead of contributing to robust, integrated analyses, scientists who use one approach have tended to stand aloof from researchers using the other approach. Within the climate change debate, for instance, the gap between the two has meant that relevant research has been bypassed and that researchers dismiss the research of one approach or the other on grounds that have little to do with climate change issues, according to Rayner and Malone (1998).

### Discussion

There are some parallels between combining participatory and analytical methods on the on the one hand, and interpretative and positivist epistemologies on the other. Although related, interpretive and participatory approaches are, however, not necessarily the same. In steps I and IV of Ridder et al. an interpretative approach is most applicable, but the authors do not address the epistemological balance of an SA in their paper. They stress the use of participatory tools, but these may be employed in a positivist way too (e.g. by asking a few multiple choice questions to stakeholders or by asking them to estimate certain parameters in a model). An interpretive approach in steps I and IV is instrumental to be reflexive about the definition of the issue and on the criteria for sustainable solutions from a diversity of stakeholder perspectives. In problem analysis, this may concern different perspectives on the meaning of sustainability, relevant values, interests and objectives, and the criteria to assess alternatives and impacts. In evaluating the assessment, different stakeholder perspectives can be included on how well and fair interests were addressed, power relations handled, etcetera. In this way, the interpretative approach and the use of participatory methods can support a self-reflective attitude towards the potential meaning of the assessment in the context of the question who benefits most from the assessment and the way sustainability is being framed in the particular assessment.

Another critique is that after identifying stakeholder perspectives, most SAs do not ask which interpretations and perspectives are the ones in power and which are the ones pushed to the margins. As such the concept of sustainability is in danger of being uncritically adopted and instrumentalised to achieve rather unreflected ends and to support specific paradigms (Banerjee 2003; Castro 2004; Newton and Freyfogle 2005). In contrast, a more reflexive sustainability assessment calls into question established or forged power-relations and questions the institutions that support and legitimize them (Spangenberg 2011).

To summarize, despite scholarly progress, SA applications in practice often still suffer from issues regarding mix of methods, process design and epistemological balance. We have argued that for an assessment to be effective, it needs to address both types of questions, and hence be reflexive about the definition of the issue and on the criteria for sustainable solutions. Instead of trying to forge immediate solutions, we will reflect on the typical ways in which SA approaches a problem. In the next section, we do this by drawing on a basic distinction in policy studies: between structured and unstructured problems.

## SA as problem structuring

### Unstructured problems

Sustainability issues appear as political or organizational problems that cannot be simply solved by standard routines and instruments. In policy studies such problems are labeled as ‘wicked’, as they typically involve a complex of interconnected factors as well as a pluralistic context, without offering clear suggestions how to address these. Clearly, to address such problems, more than one type of expertise is required. This is often the starting point for sustainability assessment, particularly non-legally prescribed, science-driven SA.

**Fig. 1:**
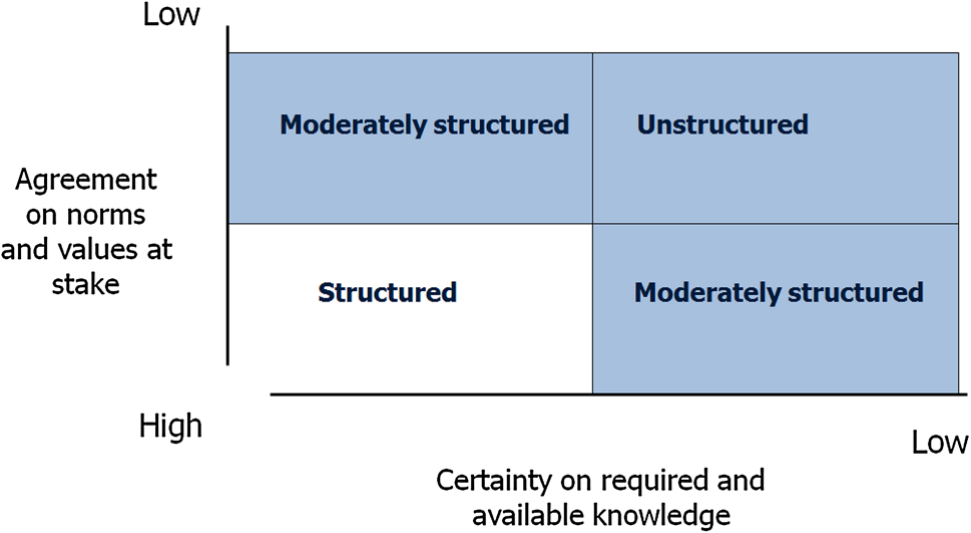
Typology of policy problems (Hisschemöller and Hoppe 1996)

In their alternative typology of policy problems, Hisschemöller and Hoppe (1996) distinguish between ‘structured’ problems and ‘unstructured’ problems, which differ in two dimensions (see Fig. 1). First, they differ in the degree in which consensus exists about norms and values: are the norms and values at stake explicitly or implicitly shared, or diverging and contested? Second, they differ in the degree in which the means (knowledge, instruments) to reach the goals are seen as available and unproblematic. In the case of structured problems, there is agreement about what has to be achieved and how it can be done. In the case of unstructured problems, both the ends and the means are contested.

Described in this way, the ‘(dis)agreement on ends and (dis)agreement on means’ typology of unstructured problems is distinct from the ‘plurality and complexity’ typology of wicked problems. Yet, they are closely interrelated, which is probably why both typologies have often been perceived as one and the same. Lack of consensus on ends or means is directly associated with the plurality in values and interests of today’s society, whereas disagreement and uncertainty about which kinds of knowledge are relevant to the solution of a problem will at least in part be caused by the growing complexity of problems.

Hoppe (2011) shows that policy makers aim to render unstructured problems structured; since there are societal pressures to deliver, policy makers and bureaucracies cannot operate on unstructured problems and thus need to make them manageable. Hence, it is in their interest to reduce the knowledge uncertainty and normative disagreement.

Arguably, SA is a means to achieve such reductions. After all, in an SA-exercise a broad range of considerations and perspectives are taken as input, and processed into knowledge claims and recommendations. That is, an SA exercise reduces the uncertainty and ambivalence of the sustainability issue and structures the problem, to some extent. The question, then, is which reductions are made by SA approaches and what is gained or lost in such moves?

### Three ways of problem structuring

**Fig. 2:**
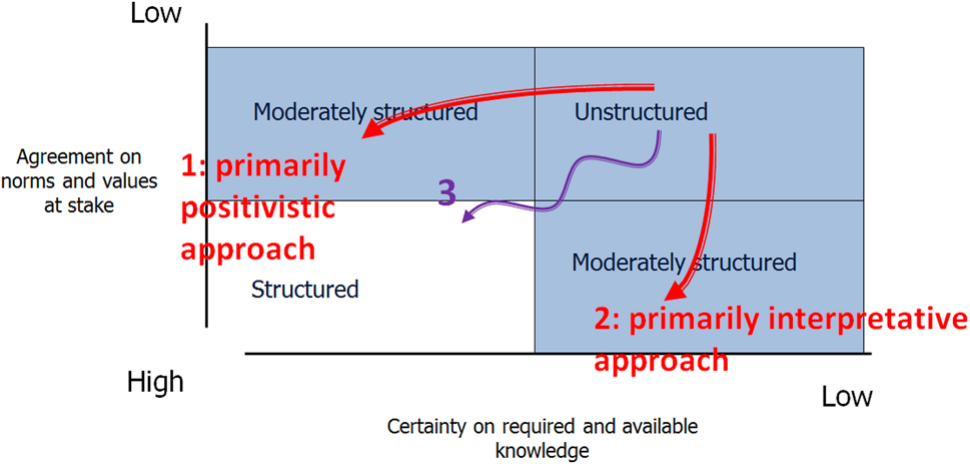
Three routes that SA approaches typically take (modified after Hisschemöller and Hoppe 1996)

Sustainability Assessments in practice have responded to this question of reductions in various ways. By reflecting on a broad range of SAs in practice, broadly three types of routes or ways can be identified in SA studies (see Fig. 2), of which we provide examples in “Examples of the three ways of problem structuring”.

Some SAs neglect the disagreement on norms and values in the first place. They elaborate more or less complex models and include input from stakeholders as a next step. Here, the normative indeterminacy is circumvented by adding stakeholder opinions or perspectives as an additional ingredient of the models. We label this as a primarily positivistic approach. In contrast, other SAs start with normative deliberations invoked by the sustainability issues and include factual analysis to enrich the dialogue. The complexity of the problem field, thus, is circumvented by framing the sustainability issue predominantly as a deliberative exercise following a primarily interpretative approach. A third route is to seek the middle ground and to address both analytical and normative indeterminacy to the same degree, for instance by proposing different steps in the SA exercise whilst alternating the mapping of stakeholder perspectives (through dialogues or discourse analysis) and analytical modeling.

In other words, we argue that, although a combination of ‘hard’ scientific analysis with stakeholder perspectives has been advocated for a few decades, and despite scholarly progress on participative approaches (e.g. Wickson et al. 2010; Reed 2008), there is a lot of room for improvement in effectuating this combination in the practice of SA informing decision-making.

## Examples of the three ways of problem structuring

To illustrate how the three typical routes work, we provide an example of each of the three ways of problem structuring. The EU Impact Assessment (IA) of the EU Energy Roadmap 2011 (described below) is an example of a primarily positivistic approach, an IA of the greenhouse gas reduction target in the Netherlands (Example 2) of a primarily interpretative approach, whilst an IA for water resources management in the Dutch Delta region (Example 3) combined positivistic and interpretative elements most explicitly (i.e. Route #3 in Fig. 2). Even though the projects in the examples had very different aims, and even though in many projects ‘problem structuring’ is not an explicit activity or process, they are relevant because they show that implicitly or explicitly an assessment always structures the problem in some direction or another. The examples, thus, are not introduced as proof, but illustrative examples of the three typical ways of problem structuring in SA. In each case, we will consider carefully how the problem is being structured and what this implies for the mix of methods, the process design and the epistemological balance.

### Example 1: EU impact assessment (IA)

In 2011 the European Commission published the impact assessment of the EU Energy Roadmap 2050. This roadmap provides a vision and trajectories towards an energy system with 80–95% less greenhouse gas emissions by 2050. The IA assesses the potential economic, social and environmental consequences of this roadmap.

The impact assessment of the Roadmap is a heavy, 192 pages report that follows the latest EU impact guidelines of 2009. Two thirds of the report consist of a well-documented, largely quantified justification of the model-based scenario analysis (i.e. assumptions and results). The modelling and related scenario analysis was performed with a model called PRIMES, an Energy-Economy-Environment model simulating the entire energy system both in demand and in supply. The PRIMES model covers the 27 EU Member States, 12 industrial sectors, various types of energy, energy using products and activities, and emissions, so the level of detail of the model is high. The timeframe of the model is 2000 to 2050 by 5-year periods; the years up to 2005 are calibrated to Eurostat data.

Consultation of stakeholders is a compulsory element of the IA, which is addressed in and about one page in the report. An online public consultation has been organized: an online questionnaire with seven questions, three open and four multiple choice. It was open from 20 December 2010 until 7 March 2011, so about two and half months, just before the publication of the IA in March 2011. Some 400 contributions, half from organisations and half from individual citizens, were received from a broad spectrum of organisations as well as citizens. The report explicitly states that ‘all of the Commission’s minimum consultation standards were met’. The questions included:

- How can the credibility of work on the transition to a low-carbon energy system in 2050 be ensured? (open)
- Which developments should be considered in the Energy Roadmap 2050? (multiple choice).
- What societal challenges and opportunities do you think are likely in Europe over the next decades as a result of changes in the EU and global energy system? (multiple choice).
- Which are the main areas which you think might need further policy development at EU level, in a 2050 perspective? (multiple choice).

There were no questions regarding what a sustainable energy system actually is. Nevertheless, many responses underlined the importance of public acceptance of new infrastructures and there was considerable divergence in opinions on the best way to decarbonise the energy sector.

In addition to the public consultation, representatives from the Directorate General for Energy and Commissioner Oettinger met numerous unspecified stakeholders individually. Also, the EU invited organizations and individuals to send reports of their scenario analysis in order to compare and test the robustness of the EU Roadmap and IA and received about 30 useful reports.^7^

After the one-page section on ‘Consultation and Expertise’, the 185 pages of the report bring up the term stakeholder another eight times, mostly referring to the scenario analyses of other organizations with which the roadmap has been compared. The term ‘dialogue’ is mentioned twice, once in the context of future recommendations.

All in all, the assessment is mostly a model-based scenario analysis with a very weak type of interaction with stakeholders.^8^ In one of the sections about the problem, the unsustainability of the current energy system is summarized, but it is unclear how this description came about. There are no references to any stakeholders or consultation in that section, and no reference to different views on the problem. Decreasing greenhouse gas (GHG) emissions is brought up as the key goal for 2050, but economic competitiveness and security of energy supply are included as well and in other parts of the report too, economic, social and environmental impact are considered.

We find the EU impact assessment study being representative for a group of studies that take a heavy quantitative approach whilst neglecting the normative plurality. The UN’s Millennium Eco-Assessment, for instance, is also conceptually strong and offers quantified, model-based scenarios, but is weaker in engagement of policymakers and other key stakeholders. In a similar vein, the IPCC review builds heavily on models, with policymakers only extensively involved in the last step when conclusions are formulated. A summary of the methods, process and epistemologies of this (and the following) examples is provided in Table 1.

**Table 1:** Summary of the methods, process and epistemologies in the three examples

	Mix of methods	Process design	Epistemological balance
Example/Route #1	Extensive quantitative, model-based scenario analysis Weak type of stakeholder interaction methods	Start: Short problem framing phase (by experts) Middle: Long phase of model development and analysis End: Short phase with consultation or communication of results	The model is to a significant extent based on scientific knowledge but includes many uncertainties that are estimated by experts Little attention is paid to the perspectives of stakeholders, and it is unclear how this shaped outcomes (if at all)
Example/Route #2	Strong stakeholder dialogue methods (including interactive backcasting) The technical knowledge provided to the participants was derived from studies/methods outside the assessment	Start: A thorough problem framing phase (although the GHG reduction target was set before SA) Middle: Phase of analysis of options (phase III in terms of De Ridder et al. 2007) neglected End: A thorough reflection (Phase IV) on all possible options to reduce GHG is made	Stakeholder perspectives were highly valued Expert/scientific knowledge was provided but did not play an important role
Example/Route #3	Participatory methods Quantitative, model-based scenario analysis (but only in project preparation phase) During the project the focus was on stimulating the contribution of local knowledge and expertise and supporting interaction and social learning between stakeholders	Start: A predefined problem description, and two broad directions for solution were also established before the start, leaving only limited space for development of more concrete options Middle: The actual focus of the project was on the phase of assessing the impacts of the options and discussing the preferred solution (phase III in terms of De Ridder et al. 2007) End: Follow-up in terms of reflection and learning was restricted to the project management team	Knowledge from scientific experts was input to project, but during the project this knowledge was significantly modified and extended with context-specific knowledge based on expertise of local practitioners The knowledge questions were formulated by the stakeholders themselves, and based on their values and interests, which were an explicit concern in the process

### Example 2: IA of the greenhouse gas reduction target in the Netherlands

The COOL-project^9^ was an academic (i.e. not policy-initiated) assessment of a (proposed) greenhouse gas reduction target in the Netherlands through stakeholder dialogues. Participants explored various ways to realize an 80% GHG emission reduction. The 80% was a working hypothesis and the willingness to explore this hypothesis was a prerequisite for the stakeholders to participate in the project. At the end of the dialogue, the participants gave a reasoned judgement on whether and how this could be done. The project included a series of workshops in which stakeholders discussed the feasibility of drastic reductions of GHG emissions in the long term; the opportunities and obstacles that would have to be overcome in order to reach such reductions; and the challenges and priorities for the short term. It included four stakeholder groups, representing four sectors: Industry and Energy; Agriculture and Nutrition; Housing and Construction; and Traffic and Transport. The four groups consisted of a heterogeneous set of stakeholders, including representatives from multinationals, small business companies, banks, unions, environmental NGOs, policymakers, et cetera. The identification and selection of these stakeholders had taken place on the basis of an extensive interview round that the project team had conducted in the preparation phase of the project with about a hundred stakeholders from different sectors of the Dutch economy. This extensive interview round enabled the project team to identify stakeholders from different networks who had rather different views on the issues of climate change and energy, and on the ‘best’ solutions to these issues.

The stakeholder dialogue in the project was organized through an interactive backcasting method. The characteristics of interactive backcasting, such as a heterogeneous group composition and a transparent procedure of the method, can in theory enable the integration of a broad range of stakeholder viewpoints, but according to Van de Kerkhof (2006) the backcasting procedure in the COOL project had a brainstorming character and did not provide methodological guidance for the integration of the outcomes. Particularly at the start of the dialogue when the participants still felt a bit uncertain and were not yet familiar with one another, they had the tendency to seek consensus. She concludes that a lack of methods to encourage argumentation in the dialogue has probably led to a priori exclusion of conflicting viewpoints.

To what extend did the study employ more scientific analysis and how did this feed into the stakeholder dialogue? The project had a ‘scientific support unit’, which provided the groups with scientific information and mainly included technical experts, as a result of which the groups mainly received information on the technical aspects of reducing GHG emissions rather than on policy and institutional aspects. As a consequence, the degree of differentiation in the selection of options was rather modest. All in all we can conclude that there was scientific input into the dialogue, but this was not an interactive process, in the sense that the stakeholder viewpoints had not fed into the scientific analysis (e.g. with specific questions) first.

### Example 3: IA for water resources management in the Dutch Delta region

The Dutch Delta Works program (1957–1997) provided safety against floods in the southwest of the Netherlands by closing off the major sea inlets. The freshwater lakes created this way have become an important source of drinking water and irrigation water for agriculture. However, the loss of estuarine dynamics also has drawbacks such as the loss of typical estuarine biodiversity and excessive growth of blue-green algae during summer. The algae make the water unsuitable for swimmers as well as agricultural use, and the bad smell has a negative impact on recreational use of the lakes. According to experts, mixing the freshwater again with salt water would be the most effective way to manage the algae problem. Therefore, the Dutch government proposed in 2004 in their ‘National Spatial Strategy’ to re-establish estuarine dynamics on a limited scale in the Delta region. However, the impact of this measure on the overall ecological quality of the area is still uncertain and it will also affect other current uses and users of the freshwater lakes, in particular the farmers.

A stakeholder engagement project was started (called ‘fundamental discussion’) to discuss how improvement of the water quality and nature value of the Delta, could be integrated with a more natural, sustainable freshwater supply for agriculture. Our description of this project is based on Hommes et al. (2009) and Vinke-de Kruijf et al. (2010). The project was managed by a consortium of four independent institutes with expertise on land and water management. The objective was to develop a shared insight and agreement about the most desirable directions for solutions. The participants included representatives from agriculture, water management, nature conservation and government. The process design of the fundamental discussion consisted of three plenary and several small-scale meetings, and the focus was on the Volkerak lake where the problems were most pressing. As input to the discussion, an overview of existing scientific knowledge was provided, including a problem description and a study on the future of agriculture in the Delta. During the first meeting, two directions for solutions were presented for discussion: making the lake salt-brackish by restoring estuarine dynamics, or maintaining the lake as a freshwater reservoir and controlling the algae with other means. The aim of the discussion was to get insight into the impacts of both types of solutions. However, the participants, in particular the farmers, were very critical about the scenario-based study on the future of agriculture and asked many context-specific questions about the proposed solutions and their consequences which the scientific experts could not answer, at least not on a short notice. Instead, the participants themselves (farmers, nature managers, local water managers) collected and provided new information based on their own specific knowledge and expertise, to assess impacts of the proposed solutions. During the small-scale meetings, the participants translated the two directions for solving the problem into five more specific options, which were put up for voting in the final plenary meeting. This resulted in two equally preferred options, one for making the lake salt-brackish combined with alternative freshwater supply from elsewhere, and one for maintaining the freshwater lake. In the end, consensus was reached on the first option, based on the consideration that the government could probably only be convinced to provide for an expensive freshwater pipeline in case all stakeholders would support this option.

In terms of problem structuring, the project was successful in both reducing the uncertainty in (instrumental) knowledge and reducing the disagreement between the stakeholders about the framing of the preferred solutions. The uncertainty in relevant knowledge was effectively addressed by allowing the participants to complement general scientific knowledge with context-specific practical knowledge based on their own expertise. The perspectives of participants, which were initially quite divergent and based on different values (socio-economic or ecological) and interests (dependent on fresh or salt water), converged over the course of the project. Although they did not become identical, in the end there was sufficient common ground to reach an agreement about the preferred solution.

Table 1 summarizes the key characteristics of the examples, typical for the corresponding problem structuring routes. The case of the EU IA was primarily a positivistic approach, with (methodologically) extensive quantitative model/scenario analysis, (process-wise) a neglected problem framing phase and long analysis phase, and (epistemologically) mostly based on expert knowledge. By contrast, the IA of a GHG reduction target was an example of a primarily interpretative approach, with (methodologically) extensive use of participatory methods, but little analysis methods (e.g. LCA, MCA, CBA, etc.) applied within the assessment, (process-wise) a thorough problem framing phase, whilst the analysis phase was neglected, and (epistemologically) stakeholder knowledge highly valued, whilst expert knowledge was provided but played a minor role. Finally, the IA for water management showed an example of an approach that combines positivistic and interpretative elements most explicitly. It combined participatory methods with analytic methods, (process-wise) comparable attention for problem framing and analysis, and (epistemologically) integration of stakeholder and expert knowledge to answer questions that emerged during the process.

## Conclusion: towards reflexive SA

The ambition of SA to operationalize sustainability for decision-making and the wicked nature of sustainability issues triggers questions that require different kinds and sources of knowledge. The complex as well as the pluralistic character of the issue has important implications for the methodological organization of a Sustainability Assessment. Traditionally, complex cause-and-effect relationships and pluralism are studied in rather distinct scientific fields, styles and methods. Cause-and-effect relationships have been studied mostly in positivistic approaches that are generally most suitable for understanding them. The range of models, indicator frameworks, life cycle analyses, etc., that have been developed for sustainability assessment clearly have their huge merits but typically neglect the diversity of perspectives among stakeholders, most notably their interpretation of the issue (problem), their contextual definition of sustainability in terms of criteria for solution strategies. Also, critical reflection on the implications of conducting the assessment implicitly from just one (dominant) perspective for the different groups of stakeholders is often absent. On the other hand, interpretative and dialogue approaches are strong in identifying the range of stakeholder perspectives, including divergence in values and interests, but are weak in highlighting their effects for economic, environmental, technical and socio-cultural processes or policy effectiveness. The complex-pluralistic character of sustainability issues requires Sustainability Assessment to combine these two rather distinct approaches and methods. So, based on a distinction of policy problems, this paper distinguished three typical ways of problem structuring, corresponding to three different ways of integrating reflexivity in the assessment: Route #1 as a poorly reflexive approach of impact assessment, Route #2 as reflexive but with poor or disconnected impact assessment, whilst Route #3 has a reflexive approach with reflexivity integrated in an impact assessment.

### What are barriers to take Route #3?

More research is needed to evaluate which route is mostly taken by SAs in public and private sectors and how, in order to corroborate and refine our conceptual contribution in this paper beyond the three illustrative examples we provided. One relevant question for such a review is: what are barriers to take Route #3? From the current body of literature and experiences we can already speculate on various answers to this question. A first one is that the assessment organizer or proponent might either adhere to a positivist or an interpretivist paradigm and simply ignores the other, consciously or unconsciously. Second, Route #3 may be found more challenging than Route #1 and 2. When facing the dual challenge of reducing knowledge uncertainties and normative disagreements at the same time (see Fig. 2), it is attractive to choose one and neglect the other, in order to keep it manageable.^10^ The process with a dual challenge has a lower degree of predictability and the question is to what extent the organizer of an SA is willing to accept this. This connects to a third possible explanation: the interest of the assessment organizer. Is this an independent party striving for the most salient and legitimate outcome for all stakeholders, or does the organizer have a preference for a certain outcome? The organizer has the power to shape the SA process, even within the boundaries of a legally prescribed SA procedure. It may well be in the interest of the organizer to neglect the value disagreements and keep the problem framing in his/her own hands. (For a comparative study that devotes explicit attention to managing power, see Clark et al. 2011).

The challenge of Route #3 also stems from the need to combine methods, which is in itself complicated, but especially for methods from different scientific styles and traditions, such as interpretative and positivistic approaches. Representatives of these two traditions will even disagree on what fruitful collaboration actually is, in terms of how to combine their methods. The positivist typically wants to add stakeholder values as a factor in his models (which upsets the interpretivist), while the interpretivist (somewhat caricatured) tends to treat positivist findings as ‘just another opinion’ next to that of the stakeholders (at least that’s how it comes across to the positivist). This will make things very hard in practice.

The challenges of taking Route #3 in addressing sustainability issues also emerge in recent papers on the methodology of sustainability science by Wiek et al. (2012) and Lang et al. (2012). Wiek et al. (2012) present a comparative appraisal of five sustainability science projects. In four of these projects, Wiek et al. (2012) identified serious shortcomings in the involvement of stakeholders and integration of their perspectives and interests, which they attributed to a general lack of advanced methodological competence and experience for dealing with these aspects on the side of the scientists. Also Lang et al. (2012) point at the need to ensure that expertise in dealing with stakeholders and their perspectives is represented in teams addressing sustainability problems. They recommend to contract professional facilitators to support the team in this. Lang et al. (2012) also highlight another stumbling block on Route #3: the different interests of academic (scientific innovation) and societal actors (problem solving), with the consequence that in scientist-driven SA there is more attention for ‘hard’ analysis of system complexity than for stakeholder perspectives. Lang et al. (2012) provide extensive methodological guidance on how to deal with this difference in interest, although probably many of these suggestions are easier said than done. Furthermore, their important principle of designing a methodological framework for collaborative knowledge production and integration is not elaborated. Despite the challenges of Route #3 as discussed, the potential of it is also observed implicitly in studies of participatory modelling (such as Voinov and Bousquet 2010), that suggest that a combination of a participatory approach with computer modeling to assess options will be generally accepted by the stakeholders as salient (relevant to their concerns), legitimate (reflecting their values and interests) and credible (in accordance with their causal beliefs).

### A way forward: reflexive sustainability assessment

Unstructured sustainability issues need to be structured in such a way that a balanced operationalization of sustainability for decision-making is achieved. The key challenge in SA is to align the methods, process and epistemology in a way that prevents spinning out to either side of Route #3, i.e. preventing over-emphasizing the disagreement on values or the lack of instrumental knowledge whilst neglecting the other. The challenge of increasing the use of Route #3 may be picked up in two ways: under the umbrella of one assessment or by combining outcomes of various assessments. The second option may be more feasible, since the methodology in one project is easily dominated by one of the two styles of science. On the other hand, combining different assessments may result in difficulties regarding interfaces in the sense of how results ‘fit’ to each other.

‘Aligning’ SA methods, process and epistemology means both to deliberately choose the most appropriate method(s) for a particular question in the SA process, as well as to achieve an appropriate balance of the different kinds of knowledge obtained in order to draw integrated conclusions, i.e. that the SA is epistemologically balanced. This dual ambition requires a map of a generic SA process connected to the most appropriate methods (such as in De Ridder et al. 2007) and an explicit (and argued) choice of methods in each step, while such a map should also include an indication of the type of knowledge that is sought (in terms of interpretative or positivistic character). The table below presents the generic map for reflexive sustainability assessment that we propose, which specifies epistemologies. Being explicit about the type of knowledge that is obtained, avoids the pitfall of simply ‘adding’ or mixing positivistic and interpretative results, and helps to make a mindful integration possible. The steps are numbered 1–4 although there is an element of iteration, between and also within the steps.

The subjective understanding that stakeholders have of the particular issue is a regular starting point of the assessment. These perspectives may be captured through open interviews, discourse analysis, ethnographic participation and observation. Subsequently, these understandings are interpreted by the SA researcher, and translated into an issue or problem scope and framing to be used during the assessment (including an argumentation for it). This includes the contextual definition of sustainability. Next, a more positivistic analysis of the problem follows, within the scope and frame set earlier. This includes logical cause and effect relationships, which may be portrayed in causal diagrams or flow diagrams, in a qualitative or quantitative way. This analysis may encounter various knowledge gaps, which can be translated into knowledge questions for the assessment. The analysis leads to the central question of the assessment. Step 1 ends with a stakeholder reflection on the previous activities and results. This may adapt the problem framing, analysis and assessment question.

The second generic step in a sustainability assessment procedure is to identify all possible options so as to deal effectively with the issue or problem as defined in Step 1. Regarding the longer time-frame of most sustainability issues, policy scenario development and analysis is a typical tool in this step. This may include stakeholders, but scenario development is primarily about explicit knowledge routed in a positivistic approach. Therefore there is need for additional methods that make stakeholder perceptions and appreciation of the generated options explicit.

The third step of a typical sustainability assessment procedure is about assessing the details of the plausible options, scenarios and/or policy interventions developed in the previous step, with the final aim of selecting options for (recommended) implementation. The emphasis typically lies on the analytical tools, such as models, indicator sets, cost–benefit analysis tools and physical analysis tools.

Depending on the context in which the SA is organized, the concluding advice may be independent, without a formal need for follow-up, or part of a policy or strategy cycle. To stimulate the usefulness of the SA, it is generally advisable to organize a monitoring and learning process in the implementation step after the SA project, which could provide feedback to the steps of option assessment and problem definition. As this step is usually outside the SA procedure, it is placed in a separate box in Table 2.

**Table 2:**
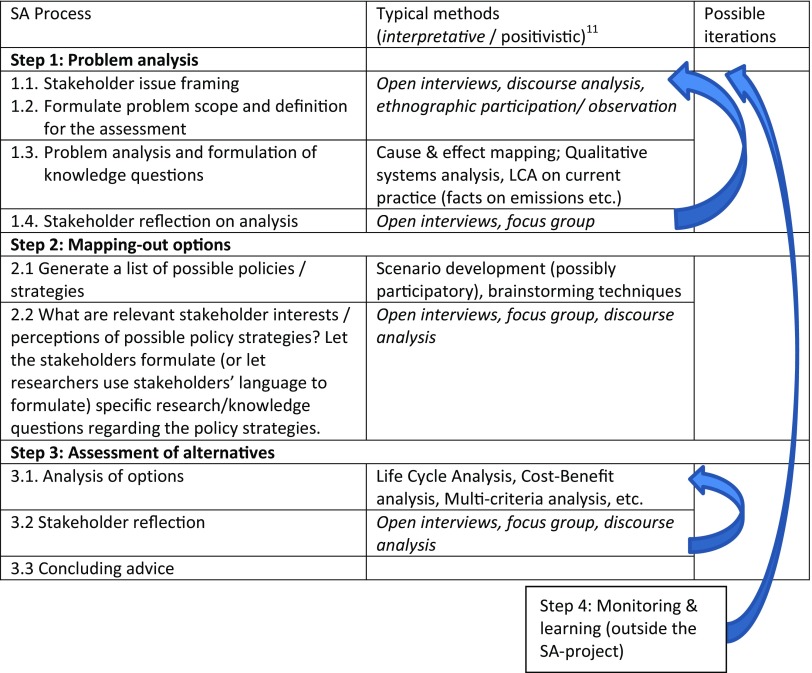
Generic reflexive SA process map

Of course, the generic character of the map means that it needs adaption for each specific case regarding which steps need to be included and which methods are most appropriate. The context specific nature of sustainability issues will continue to require customized assessment and customized outcomes. Nevertheless, our SA map builds *reflexivity* into the generic SA process, which, in combination with the other steps, can contribute to more epistemological balance of interpretative and positivistic elements. The reflexive sustainability assessment we propose occupies the middle ground between more ‘transformative’ SA approaches (such as ISA in Weaver and Rotmans 2006) on the one hand, which demand radical changes in legal and governance structures, and on the other the widely implemented but strategically less effective environmental impact assessment tradition (Morgan 2012). Reflexive SA can be implemented within existing governance structures, and, rather than just calling for more stakeholder participation, the Reflexive SA process map specifies how *reflexivity* can be built into the SA procedure.

## Footnotes

1 With ‘structuring’ we mean systematically analyzing the problem.

2 This is especially true in the impact assessment traditions where many decisions have been made before the SA commences.

3 More examples can be found in: Dalkmann et al. (2004), Devuyst (1999), Lee and Kirkpatrick (2001), Weaver and Rotmans (2006).

4 Although EIA is typically very thin on step I and II, usually assessing only one option.

5
*Interpretative* is slightly more common than *interpretive* in 21st-century British publications. Everywhere else, including in the US, Canada, and Australia, the shorter form is preferred (http://grammarist.com/usage/interpretative-interpretive/).

6 The six epistemological stances they distinguish are (1) to construct a positivist science, (2) to study systems, process, change, (3) to map contexts, (4) to understand patterns of symbolic discourse, (5) to understand how social reality is created and (6) to obtain phenomenological insight, revelation. The first is associated with the extreme objectivist approach to social sciences, the last with an extreme subjectivist approach.

7 A variety of international organisations, industry associations, individual companies, NGOs and research/academic institutions have put forward mid- and long-term energy scenarios (and names of organizations are specified in an Annex). In order to make a representative sample, 28 studies were identified by screening contributions and publications from stakeholders.

8 See Talwar et al (2011) for a typology of research regarding user engagement.

9 The COOL project lasted from January 1999 until May 2001 and was financed by the National Research Program on Global Air Pollution and Climate Change. The project included three dialogue projects, taking place at three different geographical levels: national (Dutch) (Hisschemöller et al. 2002), European (Andersson et al. 2002) and global (Berk et al. 2001). Here we report mainly on the experience of the National Dialogue.

10 Based on an analysis of six projects, Hegger et al. (2012) find that processes of co-creation between a multitude of stakeholders can be steered only to a limited extent.
